# Coupled Effects of High Temperature and Steel Fiber Content on Energy Absorption Properties of Concrete

**DOI:** 10.3390/ma17143440

**Published:** 2024-07-11

**Authors:** Ping Li, Jie Feng, Jiming Gu, Shiwei Duan

**Affiliations:** School of Mechanical Engineering, Anhui University of Technology, Maanshan 243032, China

**Keywords:** steel fiber concrete, high temperature, energy absorption capacity

## Abstract

The associated effects of temperature and steel fiber content on the energy absorption properties of concrete were examined using quasi-static uniaxial compression tests of concrete materials with varied steel fiber contents (0%, 0.5%, 1%, and 1.5%) at various temperatures (20 °C, 200 °C, 400 °C, and 520 °C). The experimental findings demonstrate that steel fibers can greatly boost concrete’s ability to absorb energy and that the toughness index rises with steel fiber concentration. The energy absorption capacity of concrete under high-temperature conditions also significantly decreases as temperature rises, and the energy absorption ability of steel fiber concrete under the same temperature is superior to that of plain concrete. The coupled influence factor K of temperature–steel fiber percentage characterizing the energy-absorbing ability of concrete was determined, and the coupled influence law of temperature and steel fiber content on the energy-absorbing capacity of concrete materials was summarized and analyzed on the basis of the experimental data of high-temperature compression. Equivalent equations for steel fiber reinforcing and temperature weakening effects when they are comparable (K = 1) are developed and equivalent parameters for concrete materials are given.

## 1. Introduction

Because of its great strength and resistance to deformation, concrete is a material that is frequently employed in construction, particularly in the engineering areas where it is utilized to build bridges and tunnels [1]. However, the destruction of plain concrete with plastic deformation reveals its very poor toughness, and under the action of a load, it is prone to brittle damage, greatly limiting the further development of concrete. To prevent the above defects, a certain amount of uniformly distributed steel fibers are mixed with plain concrete to inhibit the generation and expansion of internal cracks. It has been shown that the addition of fibers can lead to an improvement in the mechanical properties of concrete and improve its application in engineering construction [2,3]. Due to the height and spatial complexity of buildings, building materials are more likely to be affected by fire during their service life, and fire accidents can result in deaths and injuries, a significant loss of natural resources and material wealth, and in some cases, major social impacts. Fire loss statistics show that among all kinds of fires, building fires are the types of fires that occur more frequently and cause more serious losses [4,5]. When concrete structures are subjected to high temperatures, the mechanical properties of concrete are affected [6,7]. Therefore, how to improve the mechanical properties of concrete under high temperature has been the focus of scholars’ research. The ability of an engineering material to absorb energy during a load is known as toughness, and it is a crucial performance indicator in structural design. From the “law of the barrel”, it can be seen that the toughness improvement in fragile materials such as concrete plays an important role in its overall performance. The energy-absorbing capability of concrete can be used to more precisely characterize the effects of temperature and steel fiber additives on concrete. By studying the energy absorption properties of concrete, it is possible to avoid analyzing complex deformation processes and facilitate the exploration of the nature of concrete damage.

Adding fibers to concrete can inhibit the generation of internal fissures, increase the material’s toughness, and enhance the way that concrete structures deform [8,9,10,11,12,13,14,15,16,17]. Song [18] and Zhang [19] looked at steel fiber concrete’s mechanical characteristics, including its modulus of rupture, compressive strength, and toughness index. The findings demonstrated that as the volume fraction of steel fibers in concrete grew, so did its compressive strength and compressive toughness indices. Jang S. et al. performed an experimental investigation on the toughness ratio of steel fiber concrete under compressive and flexural loads [20]. The findings demonstrated that as the percentage of steel fiber grew, a rise was also observed in the flexural strength ratio and the compressive toughness ratio. Sun et al. [21] found that the dynamic mechanical properties of both plain concrete and steel fiber concrete were affected by the strain rate through dynamic tests under impact loading, and the compressive strength and toughness increased with an increase in the strain rate. Li et al. [22] found that mixing 0.9% steel fibers with concrete resulted in a slight increase in its modulus of elasticity and compressive strength, a significant increase in its flexural toughness, and a change in its damage mode from brittle fracture to ductile fracture. Yang et al. [23] studied the influence of varied steel fiber content on the mechanical characteristics and fracture toughness of concrete. The results show that the fiber volume fraction has a substantial influence on the mechanical characteristics of concrete and that increasing the steel fiber concentration improves the fundamental mechanical properties and fracture toughness of concrete. Many studies have been conducted on the toughness of concrete materials, but most of them have concentrated on showing how fibers can increase the toughness of concrete. Research on the characteristics of toughness under high-temperature exposure in concrete has been less extensive.

The high-temperature mechanical properties of steel fiber-reinforced concrete are attracting increasing attention among researchers, but most of the studies have focused on strength properties [24,25,26,27,28], largely ignoring the energy-absorbing ability of the material and structure. Lau and Anson [29] subjected plain concrete and steel fiber concrete with 1% admixture to temperatures between 105 °C and 1200 °C for compression and flexural tests. The findings reveal that the toughness of both plain concrete and concrete with 1% steel fiber content declines with rising temperature, although steel fiber concrete resists higher temperatures than plain concrete. Sukontasukkul et al. [30] conducted flexural toughness tests on plain and fiber concrete according to ASTM 1018 [31], where the load after the peak and the toughness under flexural loading increased at lower temperatures (400 °C) and then decreased with increasing temperatures (600 °C and 800 °C). Poon et al. [32] investigated the changes in compressive strength, stress–strain relationship (stiffness), and energy absorption capacity (toughness) of concrete under high temperature conditions. The findings demonstrate that while concrete loses its ability to absorb energy rather slowly when exposed to high temperatures, it loses its stiffness far more quickly than its compressive strength. When concrete is exposed to high temperatures, steel fibers significantly slow down the deterioration of the concrete’s toughness. Additionally, steel fiber-reinforced concrete can absorb more energy at high temperatures than plain concrete. The energy absorption capabilities of fiber concrete have been successfully tested at high temperatures by the aforementioned researchers, and their results show that the temperature weakening effect and the reinforcing effect of the steel fibers affect concrete’s capacity to absorb energy. The aforementioned studies, however, primarily focus on the experimental analysis of the high-temperature performance of a single steel fiber admixture. They are less concerned with the law and mechanism governing the competition throughout the reinforcing effect of steel fibers and the weakening effect of temperature on concrete’s ability to absorb energy under high-temperature conditions, as well as the law governing the impact of various steel fiber contents on concrete’s ability to absorb energy at high temperatures.

In order to more correctly explain the combined effect of temperature and steel fiber admixture on concrete, this work performs experimental research on the high-temperature compression of concrete with varied steel fiber contents under different temperature settings. The law governing how temperature and steel fiber changes affect concrete’s ability to absorb energy is also derived from this. It also determines the coupled effects of temperature weakening and steel fiber strengthening on this ability, as well as the circumstances in which each becomes dominant, and it separates the dominant regions of these two effects. Finally, the mechanisms of competition and action of these two effects during the deformation and energy absorption processes of concrete are revealed. The study’s findings can be used as a reference to optimize the structural design of steel fiber concrete at high temperatures.

## 2. Materials and Methods

### 2.1. Uniaxial Compression Experiment

The raw materials used in the preparation of the test mainly include cement, sand, stone, steel fibers, water, and water-reducing agents. The cement used is the Chaohu brand 425# slag cement (produced at Chaohu Cement Plant, Chaohu, China). It has a compressive strength of 42.5 MPa. In the mixing process, cement plays a coagulation role, reacts fully with water to form a gel, and bonds with the aggregate to set and harden into concrete. The coarse aggregate of 5–10 mm crushed stone was selected as the strongest substance in the concrete, mainly acting as an inert filler. A medium sand with a fineness modulus of 2.3 was selected as the fine aggregate. Before mixing, both coarse and fine aggregates are washed to remove the mud content. The steel fibers are ultra-fine, ultra-short, copper-coated steel fibers with a length of 6 mm and a diameter of 0.175 mm, which are mainly used for reinforcing and toughening purposes. The concrete raw materials were mixed to achieve the proportioning requirements as shown in Table 1. The specimen is a cylinder of the following size: φ 50 mm × 100 mm.

The uniaxial compression tests under static loading were carried out on an electro-hydraulic servo pressure tester model MTS810 (manufactured by MTS Corporation, Eden Prairie, MN, USA, model MTS 810) with a loading rate of 0.5 mm/min. A high-temperature heating unit was installed on the MTS material testing machine to meet the heating requirements for concrete specimens in this paper. To reduce the errors occurring in the experiment, based on the requirements of the MTS testing machine and device for the size of the specimen, the static specimen was machined into a cylinder of φ 50 mm × 100 mm, with the parallelism of the surfaces of the two ends within 0.05 mm, and the flatness of the surfaces within 0.02 mm. In this study, concrete with 0%, 0.5%, 1%, and 1.5% volume fractions of steel fibers was chosen for uniaxial static compression experiments at four temperatures (20 °C, 200 °C, 400 °C, and 520 °C). Three replications of the tests were conducted at the same temperatures for varying steel fiber contents. A detailed description of the specimen fabrication and maintenance process, specimen heating regime, and experimental set-up and procedure can be found in Li’s study [24].

### 2.2. Energy Absorption and Toughness Index

The destruction of materials can be viewed as the interconversion of different forms of energy. Each stress–strain state of concrete corresponds to a corresponding energy state during the loading process of concrete, which is a process of converting external loads into energy. Toughness properties characterize how well a material absorbs energy and resists fracture damage during deformation, and the magnitude of energy absorbed per unit volume of concrete during compression (that is, the specific energy) can be expressed in terms of the area covered by the stress–strain curve [33,34,35,36], which is expressed as follows:

(1) $$W=\int_{0}^{\varepsilon }\sigma d\varepsilon .$$

where W is the specific energy absorbed; σ denotes stress; and ε denotes strain.

**Figure 1:**
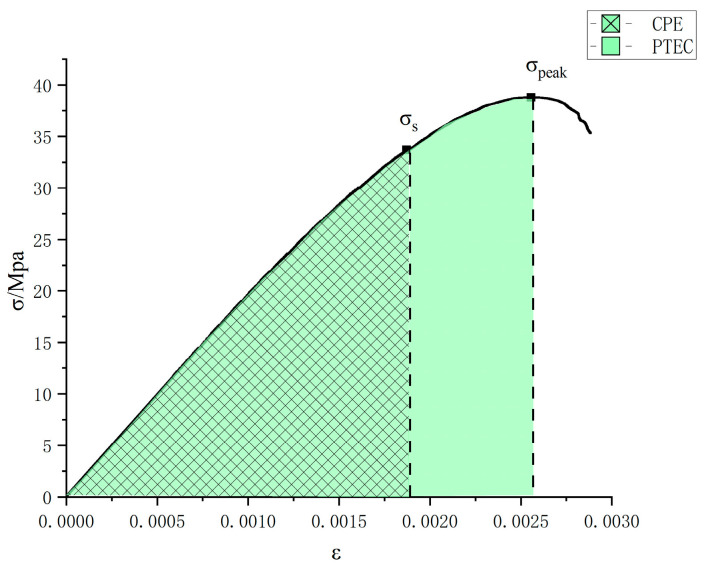
Stress–strain curve division.

The magnitude of a material’s capacity to absorb energy during deformation is measured by its toughness. Khan and Qin et al. [37,38,39,40] found that a measure of concrete’s load-bearing capacity can be expressed quantitatively. Pre-cracking compression absorbed specific energy (CPE) is the amount of energy absorbed per unit volume of steel fiber concrete due to compressive deformation prior to the appearance of visible cracks and is represented by the area below the stress–strain curve at the time the specimen reaches the yield stress ($\sigma_{s}$). The specific energy absorbed in pre-peak compression (PTEC) is the total energy absorbed per unit volume of steel fiber concrete due to compressive deformation at the time of reaching the peak stress ($\sigma_{peak}$), and its value is represented by the area below the stress–strain curve at the time of reaching the peak stress in the specimen, as shown in Figure 1.

In this paper, the energy ratio method is used as a method to describe the toughness index of concrete, which uses the ratio of the toughness at a given point to the toughness at a characteristic point to characterize the toughening effect of the material after the characteristic point [41]. According to the above definition, the compressive toughness index (CTI_S_) is the ratio of the value of energy absorbed before the peak load (PTEC) to the value of energy absorbed before the initial cracking (CPE), that is, PTEC/CPE, which reflects the toughness properties of the material when subjected to a load. The temperature toughness index (CTI_T_) is the ratio of the pre-peak load energy absorption (PTEC) of concrete at elevated temperatures to the pre-peak load energy absorption (PTEC) at normal temperatures.

## 3. Analysis of Results

### 3.1. Reinforcing Effect of Steel Fibers on Energy Absorption Capacity

**Figure 2:**
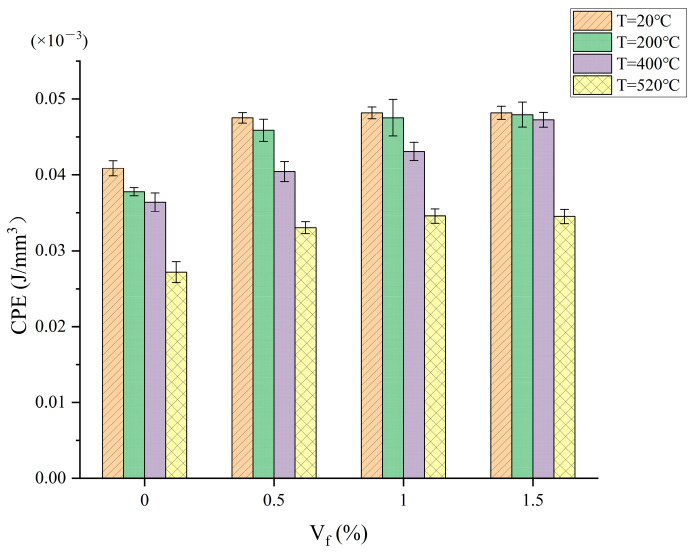
Relationship between CPE and steel fiber content.

According to Figure 2, the concrete with 0% steel fiber content absorbed the least amount of energy before cracking among all the concrete specimens. The inclusion of a particular amount of steel fibers in the concrete creates resistance between the fibers and the matrix. This increases the stiffness and ductility of the concrete. Overall, CPE rises as steel fiber concentration increases. Before cracking, the force characteristics of the specimen is mainly determined by the matrix alone, and with an increasing load, the matrix bears some of the load transfer to the fiber, the concrete in the process of elastic deformation gradually from the matrix alone to the matrix and the steel fiber joint bearing, and absorbs part of the energy.

**Figure 3:**
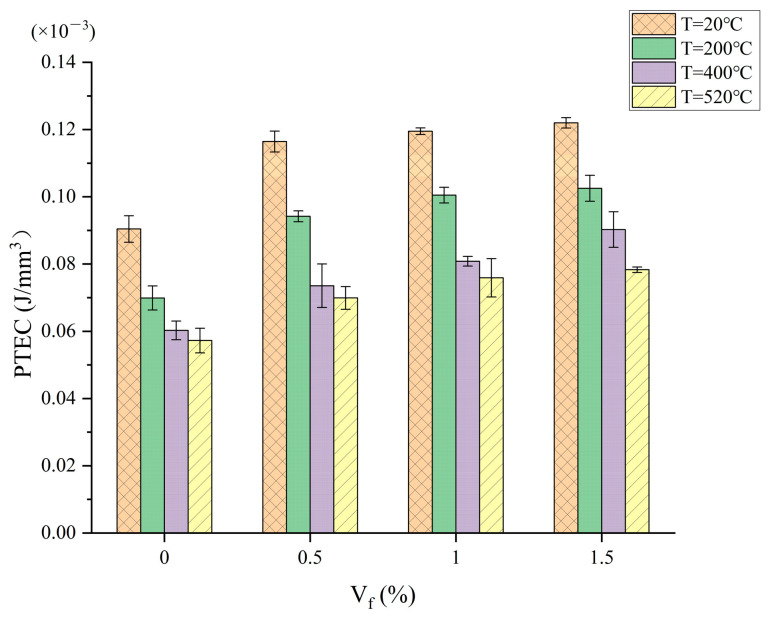
Relationship between PTEC and steel fiber content.

The destruction of concrete begins with the creation of microcracks, and as the load increases, the cracks gradually expand and are penetrated, eventually leading to the process of concrete failure. Steel fiber, as a rigid fiber, has a role in controlling the deformation of the specimen during the crack expansion and extension process. As seen in Figure 3, it is possible that the inclusion of steel fibers enhances the ability of the concrete to absorb energy as the PTEC of the steel fiber concrete specimens is significantly higher than that of plain concrete and increases as the amount of steel fiber in the concrete increases. In contrast to Figure 2, the concrete specimens’ PTEC is noticeably greater than their CPE. After the concrete cracks, it enters an elastic–plastic phase where the steel fibers spanning the cracks begin to transfer stress. As the load increases, the crack further expands, the fibers are gradually shed, the number of fibers linked across the crack gradually increases, and the ability to transfer stresses gradually increases. Concrete must absorb more energy from the environment to prevent cracks from growing larger and from developing new ones. As a result, the specimen’s capacity to absorb energy increases noticeably. A comparative analysis of CTI_S_ is provided below to help explore the reinforcing of a concrete matrix by steel fibers in more detail.

**Figure 4:**
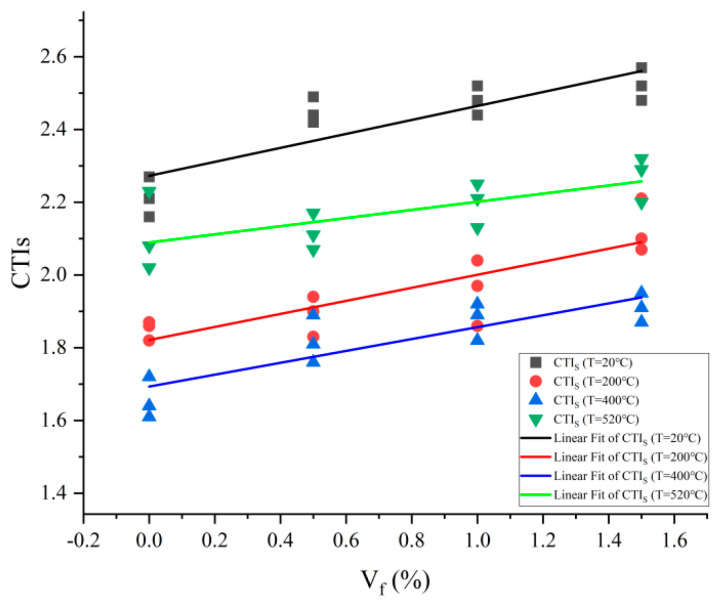
Relationship between toughness index CTI_S_ and steel fiber content.

The toughness index CTI_S_ is calculated by dividing the energy absorption value before peak loading by the energy absorption value before cracking starts. Toughness is measured by a dimensionless metric, the variation of which with steel fiber content is shown in Figure 4.

The toughness index CTI_S_ is a tool for measuring the toughness attributes of concrete that are realized during compression. When the amount of steel fiber increases, the CTI_S_ typically rises linearly. Because plain concrete crumbles quickly after the first fracture appears, its toughness index is the lowest of all. Concrete’s ductility is successfully increased by the incorporation of steel fiber, which raises the amount of energy the material absorbs during the fracture damage process. Steel fibers have a reinforcing effect on the energy absorption capacity of concrete. The CTI_S_, which progressively rises with the addition of steel fiber, serves as evidence of this.

### 3.2. Weakening Effect of Temperature on Energy Absorption Capacity

**Figure 5:**
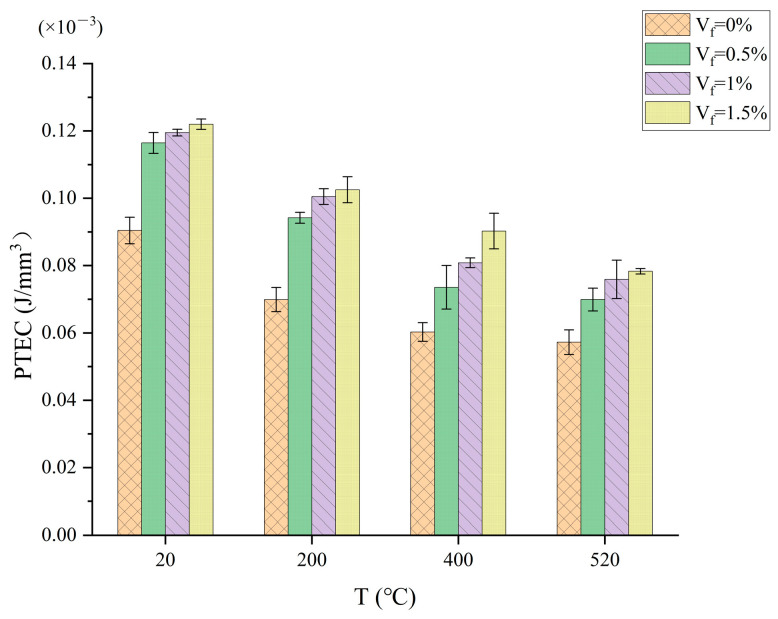
PTEC versus temperature.

Figure 5 illustrates that the PTEC decreases with increasing temperature and that the maximum amount of energy absorbed by the concrete at a normal temperature is reached. The value of energy absorbed by steel fiber concrete and plain concrete decreases rapidly when the temperature rises from 20 °C to 200 °C. After that, when the temperature rises from 200 °C to 400 °C, the value of the absorbed energy tends to decrease. When the temperature rises, the water in the matrix evaporates, the porosity gradually increases, the concrete deforms uncoordinatedly, and the degree of attachment between the steel fibers and the cement matrix is harmed, all of which contribute to the formation and growth of microcracks. In addition, the expansion of aggregates under the action of high temperatures and temperature incompatibility with the cement matrix further exacerbate the damage to the concrete, leading to a reduction in the amount of energy absorption of the concrete to the outside world. It is clear that the energy absorption capability of both steel fiber concrete and plain concrete decreases with increasing temperature and that temperature has a weakening effect on the energy absorption ability of concrete.

Although PTEC decreases with increasing temperature, steel fiber concrete always absorbs more energy than plain concrete at high temperatures. As the proportion of steel fibers in concrete grows, the amount of energy absorbed by the concrete increases rapidly. After reaching a high temperature, the change rule for the energy absorbed by concrete with different amounts of steel fiber resembles that at a normal temperature. Because steel fibers are good thermal conductors, they are mixed with steel fibers for an improvement in the overall thermal conductivity of the concrete, thereby reducing the temperature stress, so that the concrete at high temperatures can quickly achieve temperature uniformity, thereby reducing internal damage. Steel fibers can help prevent cracks from forming and growing, reduce the volume change brought on by sudden heating, and increase concrete’s ability to absorb energy.

**Figure 6:**
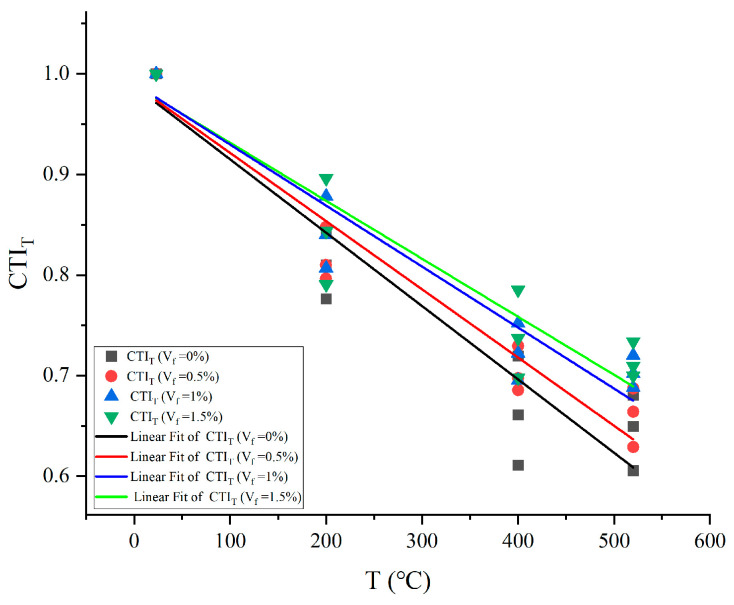
Toughness index CTI_T_ vs. temperature.

The toughness index CTI_T_ is determined as the ratio of the energy absorption value of concrete before the peak load at a high temperature to the energy absorbed by concrete before the peak load at a normal temperature, and Figure 6 shows how it changes with temperature.

The energy absorption capacity and concrete’s toughness index CTI_T_ both rapidly decline with rising temperatures. High temperatures cause microscopic or macroscopic cracks in concrete, existing temperature cracks continue to extend and expand as the load increases, high-temperature damage causes the concrete to absorb less external energy, and temperature has a weakening effect on the energy-absorbing capability of concrete. For plain concrete, as a brittle material, cracking under loading becomes easier due to the presence of its temperature cracks, and plain concrete has the lowest toughness index, CTI_T_, under different temperature conditions. A specific amount of steel fibers incorporated into concrete can effectively improve its toughness and ductility, converting plain concrete with signs of brittle damage to ductile damage. Steel fibers can also prevent internal cracks in concrete from forming and growing. In high-temperature environments, the steel fiber content of concrete causes the toughness index CTI_T_ to progressively increase, and the lowering tendency slows down as the temperature rises. Steel fiber concrete has a higher energy absorption capacity than plain concrete at high temperatures. Additionally, the more steel fibers added to the concrete, the slower the concrete’s energy absorption capacity decreases. The results indicate the ability of steel fibers to improve the energy-absorbing ability of concrete after high temperatures and to have a reinforcing effect on the energy-absorbing ability of concrete.

### 3.3. Coupled Effect of Steel Fiber and Temperature on Energy Absorption Capacity of Concrete

Compression tests were conducted on plain concrete at elevated temperatures and steel fiber concrete at normal temperature to confirm the temperature weakening effect of the concrete’s energy absorption capacity and the reinforcing effect of the steel fibers. It is yet unknown, meanwhile, how steel fibers combined with temperature can affect the ability of concrete to absorb energy. Fewer studies have been conducted on the energy-absorbing capacity of concrete, particularly when steel fibers and high temperatures are coupled. Most of the existing mechanical research in concrete focuses on the strength performance of the material. In this study, the coefficient of variation K of the energy-absorbing capacity of the combined action of temperature and steel fibers is characterized as follows, based on the experimental findings of uniaxial quasi-static compression of concrete under high-temperature conditions:

(2) $$K=W_{ST}/W_{P0}$$

where W_ST_ expresses the PTEC absorbed by a unit volume of steel fiber concrete at elevated temperatures, and its magnitude is the value of the area under the stress–strain curve of steel fiber concrete at elevated temperatures. W_PO_ expresses the PTEC absorbed by a unit volume of plain concrete at a normal temperature, and its magnitude is the value of the area under the stress–strain curve of plain concrete at a high temperature.

This factor might describe the material’s change in energy absorption capability when temperature and steel fiber are combined. In the contest between the two influencing factors, temperature weakening and steel fiber reinforcement, the steel fiber reinforcement effect wins when K > 1, that is, when the energy absorption capability of the concrete material under the combination of steel fiber and temperature is higher than that of plain concrete at a normal temperature. In the contest between the two influencing factors, temperature weakening and steel fiber reinforcement, the temperature weakening effect wins when K < 1, that is, when the energy absorption capability of the concrete material under the combination of steel fiber and temperature is poorer than that of plain concrete at a normal temperature. When K = 1, that is, when the energy-absorbing capacity of the concrete material affected by the steel fibers and temperature is equal to that of plain concrete at a normal temperature, this indicates that the concrete material is in competition with the two influencing factors of steel fiber reinforcement and temperature weakening and that the steel fiber reinforcement and temperature weakening effects are comparable to the energy-absorbing capacity of the concrete.

The association between temperature T, steel fiber content V_f_, and joint factor K using T and V_f_ as variables was examined.

**Figure 7:**
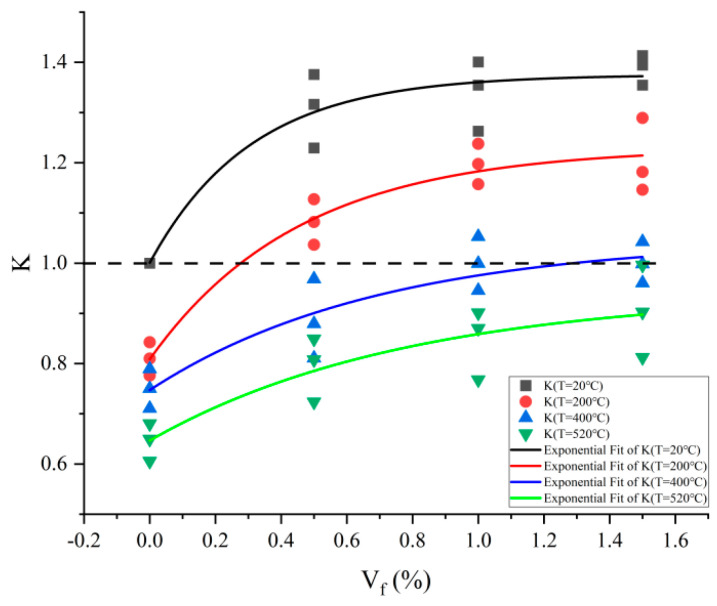
The correlation between co-factor K and steel fiber content.

**Figure 8:**
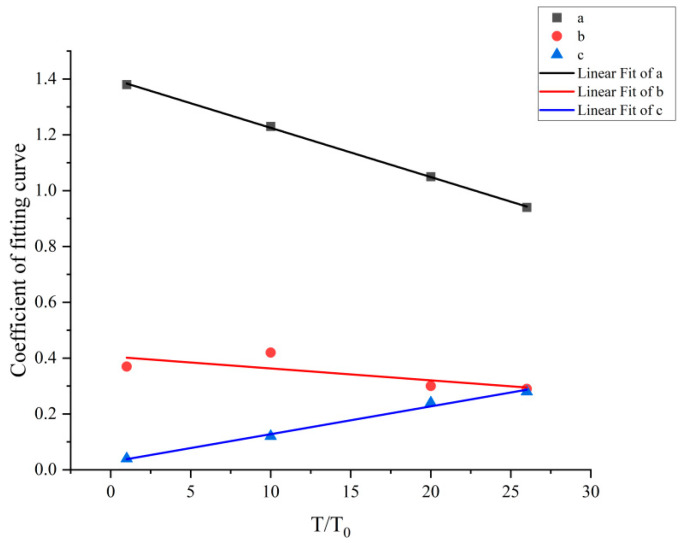
Relationship between fitting coefficients a, b, c, and temperature.

As seen in Figure 7, the change pattern of joint factor K with steel fiber content V_f_ at various temperatures is comparable to the findings of uniaxial compression experiments conducted on concrete at a normal temperature. Fitting yielded the following equation for the correlation curve between steel fiber content V_f_ and joint factor K:

(3) $$K(V_{f},T)=a(T)-b(T)*c{\left(T\right)}^{V_{f}}$$

where a, b, and c are functions of the temperature, V_f_ in %, and T in °C, and Table 2 displays the values that are obtained. Figure 8 depicts their fluctuation with temperature.

Fitting the following formula yields the relationship between a, b, and c as a function of temperature based on the experimental results:

(4) $$a=1.401-0.018(T/T_{0})$$

(5) $$b=0.406-0.004(T/T_{0})$$

(6) $$c=0.028+0.010(T/T_{0})$$

where T_0_ = 20 °C, V_f_ in %, and T in °C.

According to Figure 7, the following applies:

(1) The K-V_f_ fitting curve has a different intercept on the longitudinal coordinate axis (K-axis), which is the temperature weakening factor K_T_ (the ratio of energy absorbed under high-temperature conditions in plain concrete to that absorbed under normal-temperature conditions in plain concrete, W_PT_/W_P0_) of the material’s ability to absorb energy under normal-temperature or high-temperature conditions of plain concrete, that is, K (V_f_ = 0) = K_T_. There is a decreasing effect of temperature on the energy-absorbing capability of concrete, as evidenced by the fact that the energy-absorbing capability of plain concrete (V_f_ = 0) diminishes with rising temperatures. At a normal temperature (T = 20 °C), K > 1 was always present at different steel fiber contents, confirming that steel fibers have a reinforcing influence on the ability to absorb energy.
(2) Under the joint condition of steel fiber and temperature, when K = 1, the energy absorption capability of concrete is weakened by temperature in an amount equivalent to that of concrete that has been reinforced by steel fiber. Under this condition, the amount of energy that steel fiber concrete absorbs at elevated temperatures W_ST_ is equal to the energy absorbed by plain concrete at normal temperature W_P0_, that is, W_ST_ = W_P0_. When T = 200 °C and steel fiber percentage V_f_ < 0.28%, K < 1, indicating that the steel fiber reinforcing effect and the temperature weakening effect compete with each other as a result of the dominant temperature weakening effect on the energy absorption capability of concrete. If steel fiber content V_f_ > 0.28%, K > 1, indicating that the steel fiber reinforcing effect and the temperature weakening effect compete with each other as a result of the dominant steel fiber reinforcing effect on the energy absorption capability of concrete. When T = 400 °C, if the steel fiber content V_f_ < 1.33%, K < 1, indicating that the temperature weakening effect dominates the energy-absorbing capacity of concrete. If steel fiber content V_f_ > 1.33%, K > 1, indicating that the steel fiber reinforcing effect dominates the energy absorption capacity of concrete. When T = 520 °C, K < 1 is consistently found at varying steel fiber concentrations. The K values of concrete in high-temperature conditions are all less than the K values at a normal temperature.
(3) At different temperatures, the joint factor K shows a rising pattern as the amount of steel fiber increases, and the K-V_f_ fitting curve gradually flattens out and the slope gradually decreases, suggesting a progressive weakening of the steel fibers’ strengthening function. However, under high-temperature circumstances, the energy absorption capability of steel fiber concrete specimens was still higher than that of plain concrete. The main reason for this is the steady reinforcing effect of the steel fibers when mixed in the mortar. This reinforcing effect is due to the indiscriminately distributed steel fibers enhancing the bond between the concrete matrix and the coarse aggregate, effectively hindering the formation of cracks, allowing for uniform pressure transfer and dispersion.

**Figure 9:**
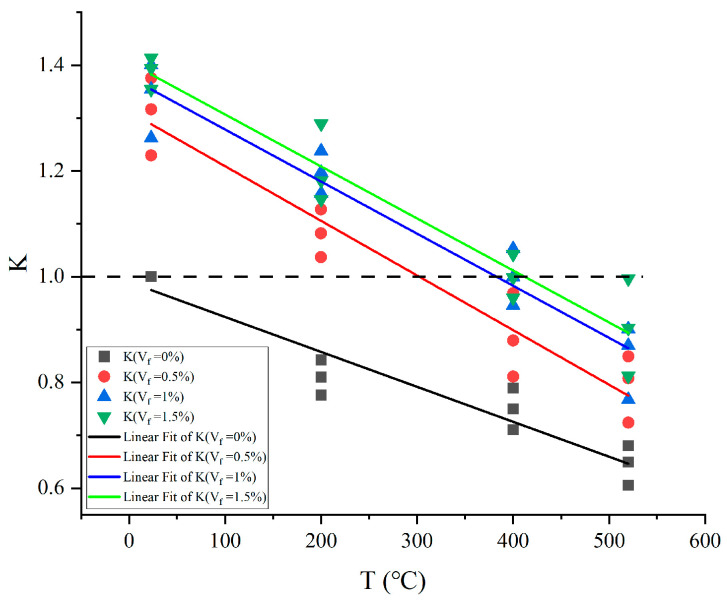
Temperature and cofactor relationships.

The variation rule of joint factor K with temperature T under different steel fiber contents is displayed in Figure 9.

**Figure 10:**
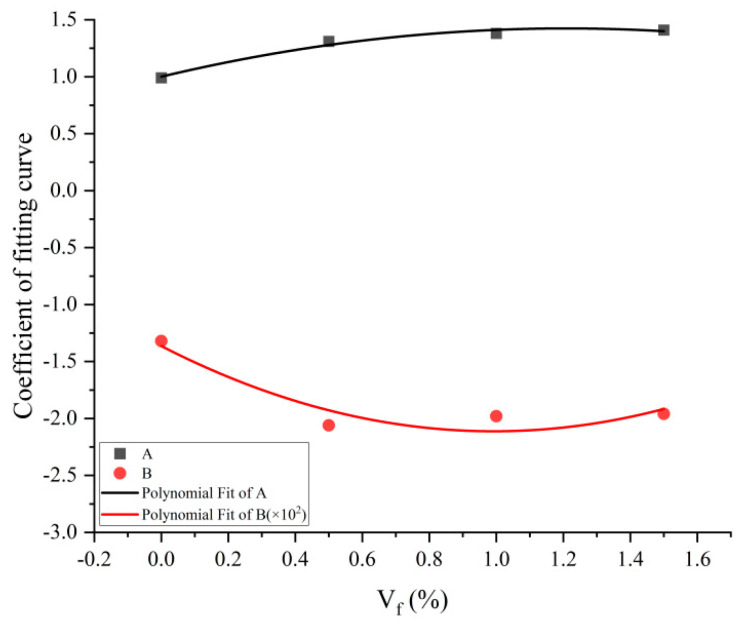
Fitting coefficients A and B and the percentage of steel fibers in relation to each other.

In order to make the equation uniform in dimension, the temperature was made dimensionless, and the equation for the relationship between joint effect factor K and the change in temperature was obtained as follows:

(7) $$K(V_{f},T)=A(V_{f})+B(V_{f})T^{*}$$

where the dimensionless parameter T* = T/T_0_, V_f_ in %, T in °C, and fitting parameters A and B are functions of steel fiber content V_f_, whose obtained values are in Table 3. Figure 10 illustrates their fluctuation with steel fiber content. (The value of B is magnified by a factor of 100 in Table 3 so that the changes in A and B can be clearly shown in the same graph).

Table 3 displays the values of the fitting coefficients A and B, and the specific expressions for A, B, and steel fiber content V_f_ (V_f_ in %) are derived from the fitting:

(8) $$A(V_{f})=1.000+0.701V_{f}-0.290{V_{f}}^{2}$$

(9) $$B(V_{f})=-1.364-1.508V_{f}+0.760{V_{f}}^{2}$$

(1) At T = 20 °C, the steel fiber reinforcing factor K_S_ is the joint factor K of temperature and steel fiber (the proportion of energy absorbed by plain concrete at a normal temperature divided by that of steel fiber concrete at the same temperature, W_SO_/W_PO_) for concrete at ambient conditions, that is, K_S_ = K (T = 20 °C), and K_S_ is a function of steel fiber content only. Under both ambient and elevated temperature conditions, the energy-absorbing capability of concrete rises as the quantity of steel fibers rises. Moreover, steel fiber concrete has a consistently better energy-absorbing capacity than plain concrete, illustrating the steel fibers’ reinforcing effect. At steel fiber content V_f_ = 0, K < 1 is always present at different temperatures, confirming that temperature has a weakening influence on the ability to absorb energy.
(2) When the steel fibers and temperature are combined, the trend of the joint factor K of steel fiber concrete with temperature is the same as that of plain concrete, and K decreases linearly with the increase in temperature. When V_f_ = 0.5%, if temperature T < 302 °C, K > 1, indicating that the temperature weakening effect and the steel fiber reinforcement effect compete with each other as a result of the steel fiber reinforcing impact on concrete’s energy absorption capability, which is dominant. If temperature T > 302 °C, K < 1, indicating that the temperature weakening effect and the steel fiber reinforcement effect compete with each other as a result of the temperature weakening impact on concrete’s energy absorption capability, which is dominant. When V_f_ = 1%, if temperature T < 382 °C, K > 1, indicating that the steel fiber reinforcing effect dominates the energy absorption capability of concrete. If temperature T > 382 °C, K < 1, indicating that the temperature weakening effect dominates the energy absorption capacity of concrete. When V_f_ = 1.5%, if temperature T < 412 °C, K > 1, indicating that the steel fiber reinforcement effect dominates the energy absorption capability of concrete. If temperature T > 412 °C, K < 1, indicating that the temperature weakening effect dominates the energy absorption ability of concrete. Despite the fact that steel fiber concrete specimens’ ability to absorb energy declines with warmth, at both normal and high temperatures, the energy-absorbing capability of steel fiber concrete is greater than that of plain concrete, and the steel fibers’ impact on the energy-absorbing capacity shows a reinforcing effect.
(3) At different steel fiber contents, it is known from the intersection of the K-T fitting curve of concrete energy absorption capacity and DIF = 1 that the higher the steel fiber percentage, the further the junction point shifts rearward, which corresponds to a higher temperature. We describe the temperature corresponding to the intersection of the K-T fitting curves with DIF = 1 for different steel fiber contents as characteristic temperature T_W_. Table 4 illustrates the relationship between steel fiber percentage and characteristic temperature.

According to the above experimental information and fitting outcomes, it can be found that the change in the concrete energy absorption capacity is not a simple superposition of the steel fiber reinforcing effect and temperature weakening effect, that is, K ≠ K_S_K_T_, and the impact of the two on the energy absorption capability are coupled with each other. The mutual coupling of the temperature weakening effect and the reinforcing effect of the steel fibers causes a change in the energy absorption capability of the concrete. K characterizes the change rule of energy absorption capability under the coupling effect of steel fiber with temperature, so K can be regarded as the coupling effect factor of concrete’s energy absorption capability.

**Figure 11:**
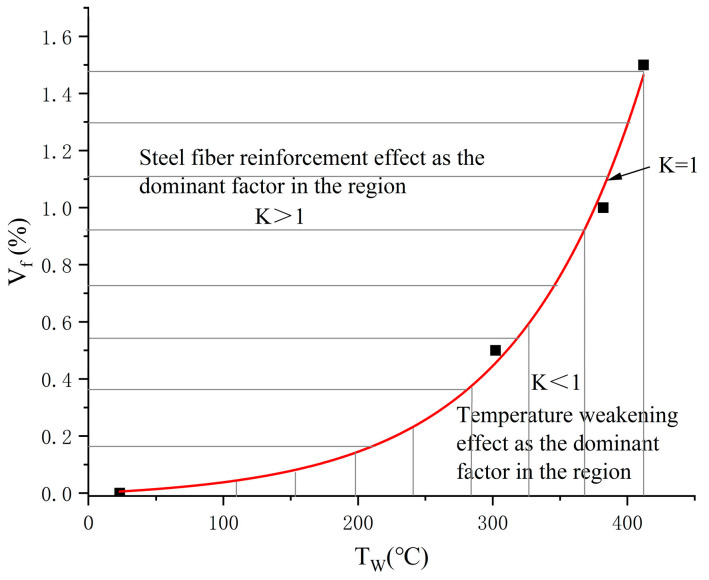
Relationship curve between characteristic temperature and steel fiber content.

To derive the inherent relationship between the energy absorption capacity’s steel fiber reinforcing impact and temperature weakening effect, it is necessary to further analyze the rule of change between coupling effect factor K, steel fiber percentage V_f_, and temperature T. According to the definition of K, when K = 1, the steel fiber reinforcing effect and the temperature weakening effect on the energy absorption capability of concrete are comparable, and the energy absorbed by concrete in the combined steel fiber and temperature condition that satisfies K = 1 is the equivalent of the energy absorption of plain concrete under the normal temperature condition. The relationship between characteristic temperature and steel fiber percentage in Table 4 was fitted and the curve obtained is shown in Figure 11. When the joint action of steel fiber and temperature falls above the curve, K > 1, at which time the material exhibits the phenomenon of reinforcement, and the energy-absorbing capacity is higher than the energy-absorbing capacity of plain concrete at a normal temperature. Conversely, the joint action of steel fibers and temperature falls below the curve with K < 1, at which point the material exhibits a weakening phenomenon, and the energy absorption capacity weakens compared to plain concrete at a normal temperature.

In order to make the equation uniform in dimension, the temperature was made dimensionless and fitted to obtain the equation relating the characteristic temperature and the steel fiber content at K = 1 as follows:

(10) $$V_{f}=a*(1-\exp\left(-b*{T_{W}}^{*}\right))$$

where the dimensionless parameter T_W_* = T_W_/T_0_, T_0_ = 20 °C, V_f_ in %, a = −2.098 × 10^−2^, and b = −0.207.

When the conditional relationship of this equation is satisfied, then K = 1, and the ability of the concrete material to absorb energy is equal to that of plain concrete at a normal temperature, that is, temperature weakening and steel fiber reinforcement compete with each other as a result of their comparable effects on the energy absorption capacity, which cancel each other out. The range below this fitted curve, that is, K < 1, suggests that the effect of temperature weakening is more effective and that the concrete material has a smaller energy absorption capacity compared to plain concrete at a normal temperature and that the concrete material in this range of steel fiber content and temperature conditions can be returned to an equilibrium curve with K = 1 by lowering the temperature or increasing the steel fiber percentage. Furthermore, in the range above this fitting curve, that is, K > 1, the impact of steel fiber reinforcement is more effective, and the energy absorption ability is greater than that of plain concrete at a normal temperature, and concrete materials in this area of steel fiber percentages and temperature conditions may be returned to the equilibrium curve with K = 1 by either decreasing the steel fiber content or increasing the temperature so that Equation (10) can be also called the content of steel fibers and the temperature of the concrete material’s equilibrium relationship equation.

### 3.4. Destruction Patterns

**Figure 12:**
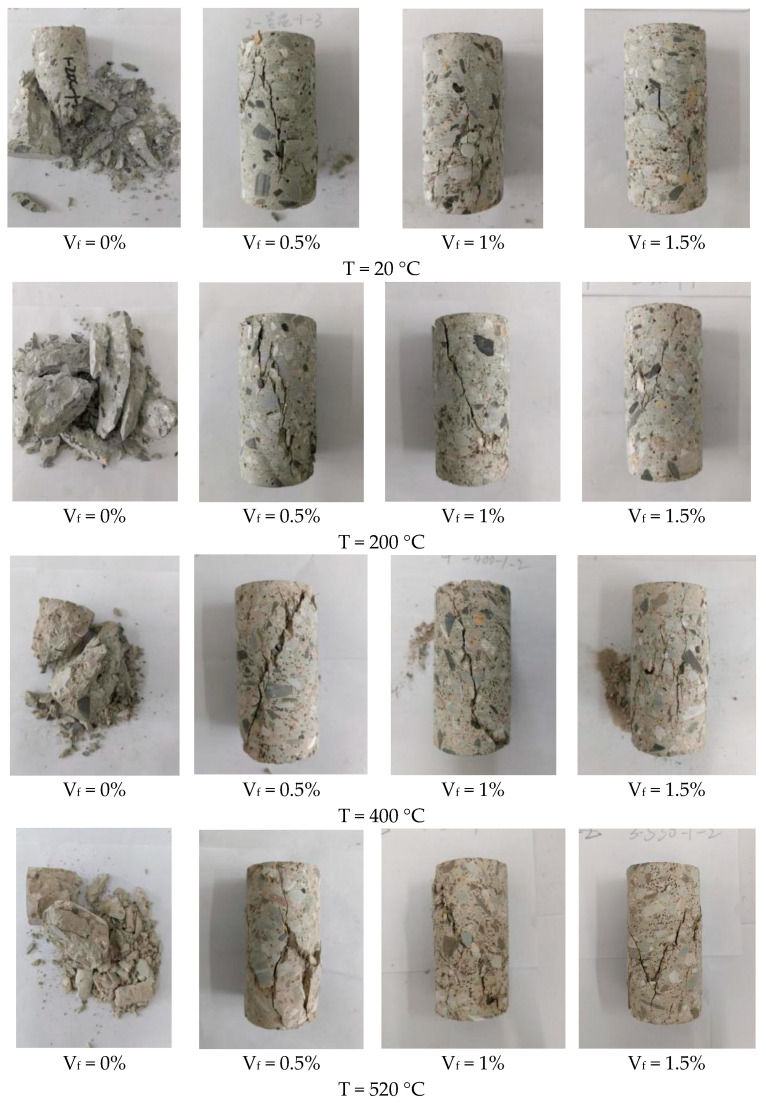
Typical damage patterns of concrete with different temperatures and steel fiber contents.

From Figure 12, the damage of plain concrete presents typical fracture characteristics of brittle materials, which occurs without significant deformation. Steel fiber concrete remains a single unit after reaching its breaking strength due to the bond between the steel fibers and the concrete. Compared to plain concrete, which breaks once it cracks, steel fiber concrete has a cracked but not scattered damage pattern, and steel fibers significantly improve the toughness of concrete. Toughness properties characterize the ability of a material to absorb energy, and steel fibers have a reinforcing effect on the energy absorption capacity of concrete. At high temperatures, the cracks in the concrete become smaller and smaller as the steel fiber percentage rises, but the damage is still severe compared to normal temperatures. This indicates that the incorporation of steel fibers can partially offset the reduction in concrete strength and fracture toughness at high temperatures, but high temperatures still have an effect on its performance, which reflects the weakening effect of temperature on the capability of concrete to absorb energy.

## 4. Mechanism Analysis

As the temperature rises, the energy-absorbing capability of both plain concrete and concrete with varying amounts of steel fiber diminishes. The high temperature effect causes water to evaporate from the concrete’s cement matrix, the porosity becomes larger, the disparity in temperature between the interior and exterior makes the external expansion larger than the internal expansion and produces tensile stresses, and cracks arise and continue to expand. Since plain concrete is a brittle material, in the presence of temperature cracks, damage occurs more easily and the energy absorption capacity decreases faster as the load increases. For steel fiber concrete, because of the effects of high temperatures, the deformation of the steel fibers and the cement mixture is not in coordination, which weakens the bonding between the two and further reduces the energy absorption capability of the steel fiber concrete.

The energy absorption capacity of concrete tends to be increased with increasing steel fiber content, both at ambient and high temperatures. As a rigid material with a high elastic tensile modulus and excellent tensile properties, the incorporation of steel fibers into plain concrete can enhance the toughness of the concrete and improve the damage pattern of concrete. When steel fiber concrete is exposed to a load, the force characteristics of the specimen gradually change from a single load bearing of the concrete matrix to a joint load bearing of the matrix and steel fibers. In addition, steel fiber also has good thermal conductivity, so the differential in temperature between the interior and exterior of the concrete is reduced, and the temperature uniformity is achieved faster, preventing the occurrence of volumetric expansion and reducing internal damage. As a result, steel fiber concrete does not immediately break down at high temperatures but continues to increase in stress as cracks steadily develop. The frictional resistance when the fibers are pulled or broken is increased by the bond between the steel fibers and the matrix, which increases the significance of the steel fibers’ ability to arrest fractures and requires more energy to be absorbed before the cracks may deepen and spread. To a certain extent, steel fiber makes concrete less brittle, and it exhibits the destructive traits of breaking but not cracking, greatly increasing the material’s ability to absorb energy.

## 5. Conclusions

In this experiment, uniaxial compression tests were performed on concrete at various temperatures (20 °C, 200 °C, 400 °C, and 520 °C) with varying steel fiber contents (0%, 0.5%, 1%, and 1.5%). This study looks into how temperature and the amount of steel fibers in concrete affect the material’s capability to absorb energy. The following are the principal conclusions:

(1) The incorporated steel fibers have a crack-blocking and toughening effect, which significantly improves the energy-absorbing capacity of the concrete, which increases as the content of steel fibers in the concrete increases. Additionally, steel fiber concrete has better energy absorption properties than plain concrete, demonstrating that the concrete’s steel fiber composition amplifies its ability to absorb energy. High-temperature action causes damage to the cement matrix and aggregate of concrete, water evaporates, porosity increases, and microcracks gradually arise and expand. As the temperature rose, the energy-absorbing capability of concrete with varying steel fiber contents all declined, which reflected the weakening effect of temperature on the energy-absorbing capacity of concrete.
(2) Under the joint condition of steel fiber and temperature, the expression of joint factor K with temperature T is $K(V_{f},T)=a(T)-b(T)*c{\left(T\right)}^{V_{f}}$, where a(T), b(T), and c(T) pertain to the temperature function, which is only related to the magnitude of the temperature. At V_f_ = 0, the joint factor K of steel fiber and temperature is the temperature weakening factor K_T_ (the ratio of energy absorbed under high temperature conditions of plain concrete to the amount of energy absorbed under normal temperature conditions of plain concrete, W_PT_/W_P0_) of the energy-absorbing capacity of the material under normal or high temperature conditions, that is, K (V_f_ = 0) = K_T_.
(3) Under the joint condition of steel fiber and temperature, the expression for joint factor K and steel fiber content V_f_ is $K(V_{f},T)=A(V_{f})+B(V_{f})T^{*}$, where A(V_f_) and B(V_f_) are functions of the steel fiber percentage, the magnitude of which is related only to the steel fiber content. At T = 20 °C, the joint factor K of steel fiber and temperature equals the steel fiber reinforcement factor K_S_ (the ratio of energy absorbed by steel fiber concrete at a normal temperature to that absorbed by plain concrete at a normal temperature, W_S0_/W_P0_) for steel fiber concrete at a normal temperature, that is, K_S_ = K (T = 20 °C).
(4) On the condition of steel fiber temperature coupling, the dominant regions of influence of the reinforcing effect of steel fiber and the weakening effect of temperature on the energy absorption capacity of concrete are given, and the intrinsic relational equation between steel fiber and temperature, when the steel fiber–temperature effect is comparable, is established as $V_{f}=a*(1-\exp\left(-b*{T_{W}}^{*}\right))$.

## Figures and Tables

**Table 1:** Raw material mix ratio of steel fiber concrete specimens.

Steel Fiber Volume Fraction (%)	Cement (kg/m^3^)	Sand (kg/m^3^)	Aggregate (kg/m^3^)	Water (kg/m^3^)	Superplasticizer (kg/m^3^)	Steel Fiber (kg/m^3^)
0	425	600	1132	184	8	0
0.5%	425	600	1132	184	8	39
1%	425	600	1132	184	8	78
1.5%	425	600	1132	184	8	117

**Table 2:** Values of fitted parameters for various temperature conditions.

T/°C	a	b	c
20	1.38	0.37	0.04
200	1.23	0.42	0.12
400	1.05	0.30	0.24
520	0.94	0.29	0.28

**Table 3:** Parameter fitting for varying steel fiber percentages.

V_f_/%	A	B × 10^2^
0	0.99	−1.32
0.5	1.31	−2.06
1	1.38	−1.98
1.5	1.41	−1.96

**Table 4:** Relationship between characteristic temperature and steel fiber content.

T_W_/°C	V_f_/%
20	0
302	0.5
382	1
412	1.5

## Data Availability

Data are contained within the article.
